# Supplementary material for: Boosting the Initial Coulomb Efficiency of Sisal Fiber-Derived Carbon Anode for Sodium Ion Batteries by Microstructure Controlling
Source: Nanomaterials (Basel). 2023 Feb 26;13(5):881. doi: 10.3390/nano13050881 (PMC10005348; doi:10.3390/nano13050881)
Supplement: Supplementary file 1 [file nanomaterials-13-00881-s001.zip › nanomaterials-2232104-supplementary.pdf]

Supporting Materials

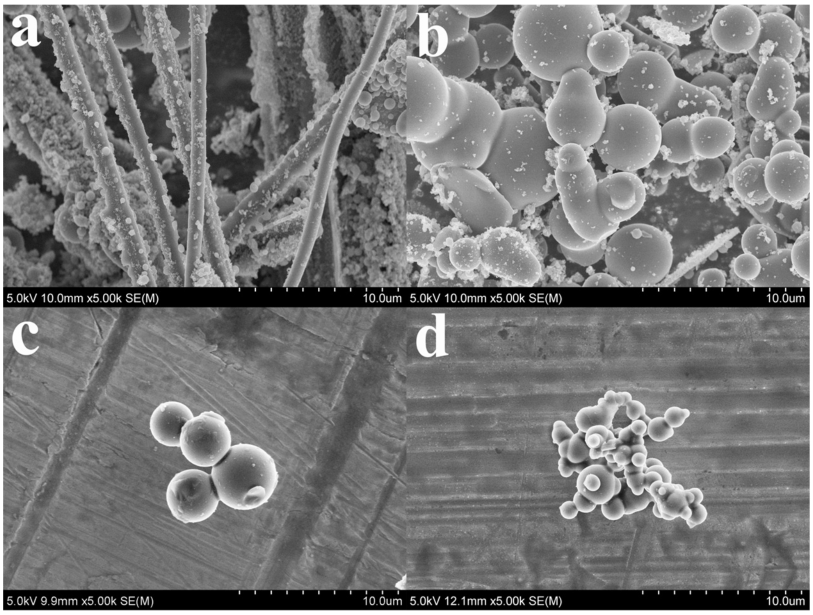

Figure S1. SEM images of (**a**) GSFC-0.1, (**b**) GSFC-1, (**c**) GSFC, and (**d**) GSFC-4.

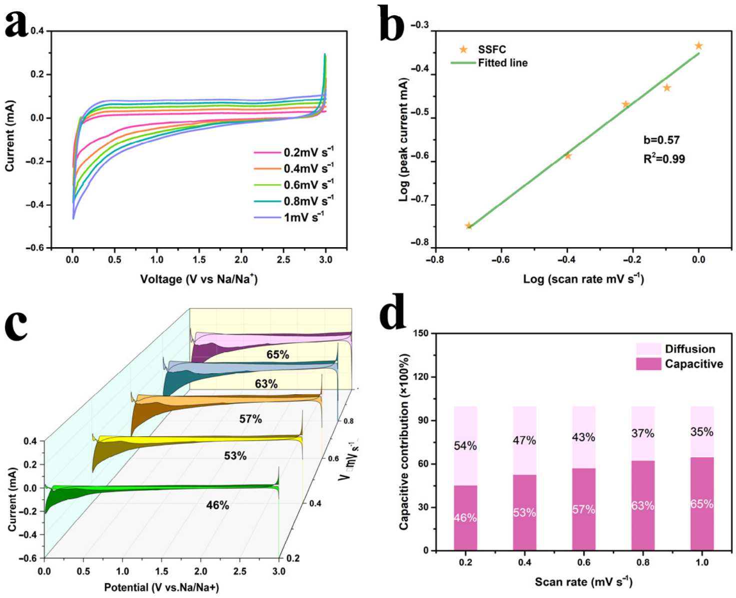

Figure S2. (**a**) Voltammetry curves of SSFC at different scan rates between 0.2 and 1 mV s^{-1}; (**b**) the plots of log(i) versus log(v) of SSFC; (**c**) pseudocapacitive contribution waterfall diagram of SSFC; (**d**) pseudocapacitive contribution of SSFC at different scan rates between 0.2 and 1 mV s^{-1}.

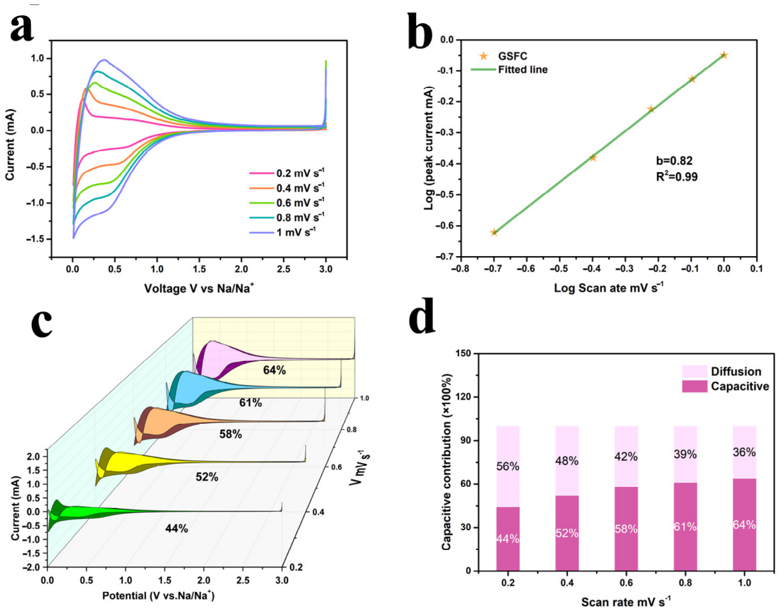

Figure S3. (**a**) Voltammetry curves of GSFC at different scan rates between 0.2 and 1 mV s^{-1}; (**b**) the plots of log(*i*) versus log(*v*) of GSFC; (**c**) pseudocapacitive contribution waterfall diagram of GSFC; (**d**) pseudocapacitive contribution of GSFC at different scan rates between 0.2 and 1 mV s^{-1}.

Table S1. N_2 adsorption/desorption isotherms parameters for the samples.

Samples	SBET (m^2 g^{-1})	Vt (cm^3 g^{-1})	$\overline{d}$ (nm)
TSFC	426.02	0.049	2.79
SSFC	1640.46	0.817	2.73
GSFC	365.24	0.061	2.77

Table S2. The comparison of the electrochemical performance (Coulomb efficiency) of TSFC with other biomass hard carbons for SIB.

Biomass Precursors	Microstructure	Current Density (mA g^{-1})	Initial Coulombic Efficiency (%)	Ref.
Sugarcane	Sheet porous	50	63	[48]
Tamarind	Bulk	50	70.4	[49]
Natural parasol fluff	Tubular	50	49.7	[50]
Spartina alterniflora	Bulk porous	50	67	[51]
Tangerine peel	Bulk porous	50	14	[52]
Camellia seed	Uneven granular	50	71.6	[53]
Sisal fiber	Hollow tube	50	76.7	This Work
